# Modelling Successful Self-Management in Adults With Cystic Fibrosis: Vicarious Self-Efficacy From Videos of ‘People Like Me’

**DOI:** 10.7759/cureus.26511

**Published:** 2022-07-02

**Authors:** Marlene Hutchings, Susan Kirkpatrick, Madelynne A Arden, Sarah J Drabble, Chin Maguire, Hannah Cantrill, Pauline Whelan, Zhe H Hoo, Martin J Wildman

**Affiliations:** 1 Sheffield Adult Cystic Fibrosis Centre, Sheffield Teaching Hospitals NHS Foundation Trust, Sheffield, GBR; 2 Health Experiences Research Group, University of Oxford, Oxford, GBR; 3 Centre for Behavioural Science and Applied Psychology, Sheffield Hallam University, Sheffield, GBR; 4 School of Health and Related Research (ScHARR), University of Sheffield, Sheffield, GBR; 5 Centre for Health Informatics, University of Manchester, Manchester, GBR; 6 Cystic Fibrosis, University of Sheffield, Sheffield, GBR

**Keywords:** cystic fibrosis, self-management, self-efficacy, health behaviour, medication adherence

## Abstract

Background

Self-efficacy is an important determinant of treatment adherence, and peer modelling of success can provide vicarious self-efficacy. A series of patient stories (‘talking heads’ videos) were developed with people with cystic fibrosis (CF) as part of the CFHealthHub multi-component adherence intervention, aiming to demonstrate success with daily therapy in ‘people like me’.

Methodology

One-to-one semi-structured interviews exploring patients’ experiences, barriers and facilitators of nebuliser adherence were audio and video-recorded between October 2015 and August 2016. Interview transcripts were reviewed to identify descriptions of problem-solving and sustained treatment success. Positive stories potentially providing vicarious descriptions of success were selected as video clips.

Results

In total, 14 adults with CF were recruited from five UK CF centres. Each participant contributed a median of five (interquartile range: 3-6) video clips, and a total of 57 unique clips were uploaded onto the CFHealthHub digital platform. Nine of those clips spanned two categories, hence, there were 66 clips across 16 categories.

Conclusions

The videos were well received though some adults were concerned that comparisons with peers might create anxiety by highlighting the possibility of future decline or current relative underperformance. It is important to sensitively support choice when providing resources aiming to increase vicarious self-efficacy. Our experience may guide the development of similar videos for people with other long-term conditions.

## Introduction

Cystic fibrosis (CF) is a genetic condition where thick mucus in the lungs and digestive system leads to progressive lung damage and malabsorption of nutrients. It is an archetypal long-term condition; there is as yet no cure for CF but highly efficacious medications exist. However, medication adherence is low in the range of 35-50% among adults with CF [1,2]. Developing effective adherence interventions will address an important unmet need [3,4] and is one of the top 10 CF research priorities [5].

The CFHealthHub digital Learning Health System (https://www.cfhealthhub.com/, ISRCTN14464661) is an improvement collaborative among 17 UK adult CF centres in which technical, behaviour change and implementation science approaches are explicitly used to learn and improve. Within a learning health system, patients can contribute new knowledge because data on all patients are routinely captured as an integral by-product of care delivery, and the capture of patient experience and best practices are seamlessly embedded in the care delivery process. At the heart of the Learning Health System is a tailored multi-component intervention to help people with CF master self-care with inhaled therapies using a digital platform supported by healthcare professionals. This multi-component intervention [6], called the CFHealthHub, used the eFlow Technology nebulisers with eTrack data-logging controllers (PARI Pharma GmbH, Starnberg, Germany) to capture objective adherence data. This was displayed for participants and interventionists on the CFHealthHub website and app. Interventionists underwent training and assessment (face to face and via Blackboard virtual learning environment [VLE]) enabling them to use CFHealthHub resources and behaviour change techniques to support participants with nebuliser adherence.

A 19-centre randomised controlled trial (RCT) showed that the CFHealthHub intervention achieved higher objectively measured adherence and lower perceived treatment burden sustained up to 80 weeks [7]. The CFHealthHub conceptual framework extends the Capability, Opportunity and Motivation (COM-B) model [8], which posits that capability, opportunity and motivation are necessary factors to perform any behaviour and create the habits of self-care.

Each of the COM-B domains may be subdivided, for example, motivation may be automatic (e.g. habit of using nebuliser) or reflective (e.g. beliefs about treatment). Self-efficacy, or a person’s perception of his/her capabilities to perform a behaviour [9], is a crucial component of reflective motivation. People with long-term conditions and high patient activation (knowledge, skills and self-efficacy) use less unscheduled care [10]. The lack of self-efficacy is an important barrier to CF medication adherence [11].

Self-efficacy can be increased through the sharing of vicarious experiences of success provided by social/peer role models because seeing people similar to ourselves succeed raises our beliefs that we can also succeed [9]. For example, if a person with CF is about to go to university watching a video of a person like them who found ways to sustain self-care while at university can increase confidence that ‘if others who are like me could succeed, I can also succeed’.

The CFHealthHub group, therefore, worked with the University of Oxford Health Experiences Research Group (HERG) to develop a set of ‘talking heads’ videos as part of the CFHealthHub multi-component intervention. HERG collects patient experiences of care pathways to familiarise others with the treatment journey. In the collaboration between the CFHealthHub group and HERG, the focus was on the challenge of the success of preventative self-care. The ‘talking heads’ videos aim to increase vicarious self-efficacy in success with daily inhaled treatments by showing a range of different types of people with CF managing or increasing their adherence. Thus, the videos provide a library of strategies used for successful self-care. This paper describes the method of selection and production of the ‘talking heads’ videos.

## Materials and methods

**Figure 1:**
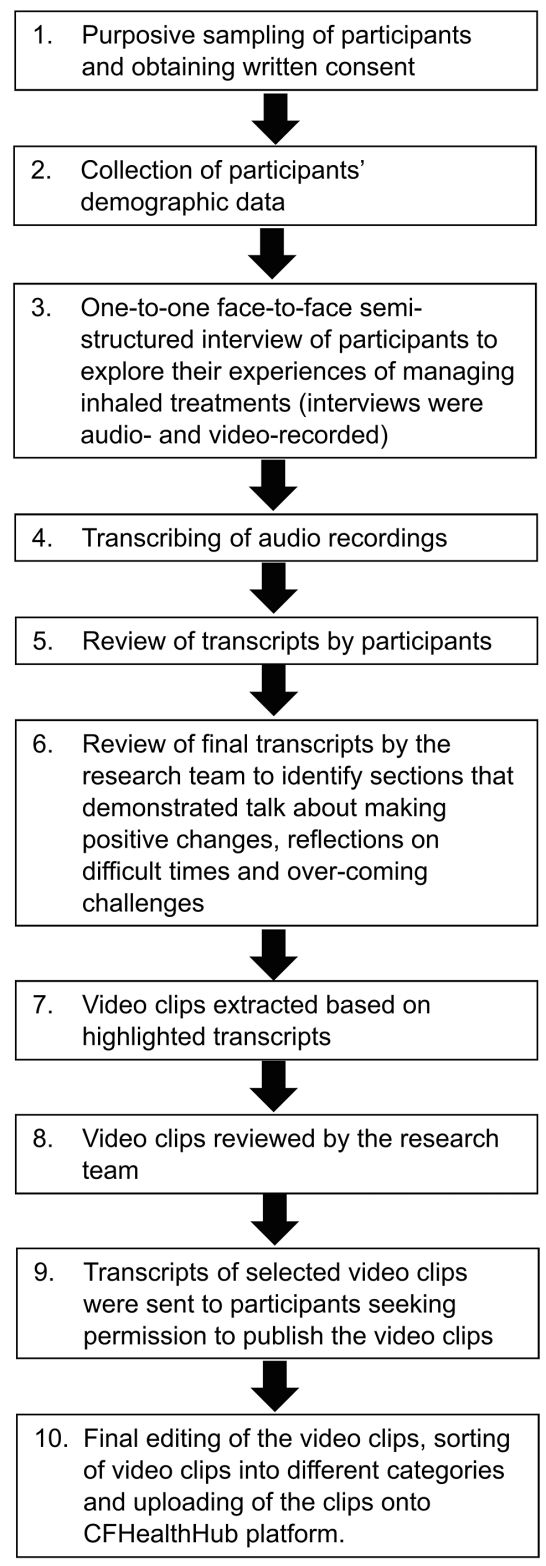
A schematic representation of the methodology to develop the ‘talking heads’ videos.

The methodology used to develop the ‘talking heads’ videos is summarised in Figure 1. Semi-structured interviews to explore patients’ experiences of managing inhaled treatments were audio and video-recorded. Regulatory approval for this study was obtained from London - Camden & Kings Cross NHS Research Ethics Committee (reference number: 15/LO/0944).

Purposive sampling was conducted using the CF registry and clinical records to identify people with CF aged ≥16 years who fulfilled the inclusion criteria (Table 1). Clinical care teams in seven UK adult CF centres provided potential participants with a participant information sheet during clinic or by post. The target recruitment was 15-20 people.

**Table 1:** Inclusion and exclusion criteria of the study.

Inclusion criteria
Diagnosis of cystic fibrosis and age ≥16 years
Reasonably stable objective adherence of >60% or sustained increase in objective adherence >2 months
Sufficiently self-aware to understand and describe their strategies to sustain adherence or what prompted their efforts to improve adherence
Exclusion criteria
Pregnancy
Post-lung transplantation
On an active lung transplant list
In the palliative phase of their disease

Participants provided written informed consent prior to data collection, including permission for audio and video recording. Demographic data were obtained from patient notes. One-to-one, face-to-face interviews lasting 45-60 minutes were performed by a HERG qualitative researcher (SK) in participants’ homes between November 2015 and August 2016. The topic guide (Appendix A) was designed to explore participants’ experiences of living with CF, to reflect on times when nebuliser adherence had been lower, and how and why the use of nebulised medication subsequently improved. Data were collected and analysed concurrently. Following each interview, audio recordings were transcribed verbatim by a HERG transcriber. The interview transcripts were sent to participants who had the option of removing any sections that they wished prior to analysis.

Final transcripts were reviewed by the CFHealthHub research team (MH, MA, SD, CM, HC, MW) to identify sections that demonstrated talk about making positive changes to adherence, reflections on difficult times and overcoming challenges or examples of problem-solving that enabled sustained adherence. The research team focused on positive stories with the potential to provide vicarious descriptions of success likely to increase self-efficacy. The sections chosen were relatively short so that the resultant videos were not too time-consuming to watch. Highlighted transcripts were then sent back to SK for HERG to extract relevant video clips from video recordings.

The research team reviewed the extracted clips. Transcripts of clips considered suitable for inclusion were sent to participants seeking permission to publish the video clips on the CFHealthHub digital platform. Participants had the option of being identified by their first names in the video library or using a pseudonym. Following confirmation of the agreement, a website delivery manager from HERG did the final editing of selected video clips and uploaded them onto the CFHealthHub platform.

The main content of the selected video clips, for example, ‘advice to younger self’ was identified and used to tag the episode in CFHealthHub. The research team (which included a person with CF) identified gaps in the ‘library’, and purposive sampling was used to try to include as yet missing experiences identified as important, such as managing CF whilst at university or as a parent caring for a young child.

The research team sorted the final version of the video clips into different categories to organise the CFHealthHub digital platform video library. For ease of use, the categories were searchable within the digital platform, and each clip also had searchable hashtags, for example, #uni alongside the video thumbnails. The video library was reviewed by the Patient & Public Involvement group of CFHealthHub, who suggested short descriptions for each video clip to accompany the thumbnails. The video clips are only available for participants of the CFHealthHub digital Learning Health System (https://www.cfhealthhub.com/), who can access the video clips by logging onto the CFHealthHub website or app.

## Results

Among the 14 participants, six (42.9%) were females, and the median age was 27 years (interquartile range (IQR): 25-33 years) (Table 2). There were 66 indexed video clips based on a total of 57 unique ‘talking heads’ video clips, with nine of those clips spanning two categories. There were 16 categories for the video clips. The numbers of video clips and the number of views from the CFHealthHub website during the ACtiF RCT for each category are summarised in Table 3. Each participant contributed a median of five video clips (IQR: 3-6 clips). One video clip initially selected based on transcript review was later omitted following a viewing. The main strategies identified by participants to help support sustained nebuliser use are summarised in Table 4. Further descriptions of the videos are in Appendix B.

**Table 2:** Demographics of the participants (n = 14).

Demographic parameter	
Age in years, median (range)	27 (20 to 57)
Gender	
Female, n (%)	6 (42.9)
Male, n (%)	8 (57.1)
Ethnicity	
White British, n (%)	13 (92.9)
White – others, n (%)	1 (7.1)
Employment status	
Employed, n (%)	11 (78.6)
Full-time carer, n (%)	1 (7.1)
Unemployed, n (%)	2 (14.3)
Education level	
Post-graduate, n (%)	1 (7.1)
Degree, n (%)	5 (35.7)
Secondary school, n (%)	3 (21.4)
Unknown, n (%)	5 (35.7)
Number of dependents	
1, n (%)	13 (92.9)
0, n (%)	1 (7.1)
Referring site	
King’s College Hospital London, n (%)	4 (28.6)
Manchester, n (%)	3 (21.4)
Oxford, n (%)	2 (14.3)
Sheffield, n (%)	4 (28.6)
York, n (%)	1 (7.2)

**Table 3:** Different categories of the video clips and the number of views from the CFHealthHub website during the ACtiF RCT for each category, * The number of views via the CFHealthHub app is unavailable.

Categories	Number of video clips	Number of views from the CFHealthHub website* during the ACtiF RCT
Advice to younger self	3	27
Coping with feeling low	3	41
Juggling treatment and life	14	187
Going to university	3	18
The importance of nebulisers	10	85
Keeping motivation up	7	77
Having a routine	7	15
Finding support	2	8
Nebuliser tips	1	10
Having a normal life	6	89
Being normal	1	9
Advice to others	2	26
Late diagnosis	1	8
Growing up with cystic fibrosis	3	12
General tips	1	4
Talking to others	2	6

**Table 4:** Strategies identified by participants to help with adherence. * Disclosing cystic fibrosis symptoms and diagnosis is associated with higher social support, social functioning and medication adherence self-efficacy whilst limited disclosure may have a negative impact on psychosocial outcomes [12].

Strategies
Be open and honest with yourself about how you are coping
Do not be afraid to ask for help, and talk to your healthcare team
Do not hide the fact you’ve got cystic fibrosis from others*
Build a routine that works for you and fits in with your own life
Set goals and gain satisfaction from achieving them
Note down the differences you feel when you are adhering well compared with when you are not
Seeking support from other people, e.g. friends, family or your healthcare team
If things are difficult, focus on some short-term goals
Think about how you want your life to be in the future, both short and longer-term
Keep your nebuliser charged; get into the routine of charging it after every use
Do not underestimate your illness or take it for granted that you’ll always feel healthy
You are the person who is in control, it is your responsibility to keep yourself well so make changes to ensure you stay as healthy as you can

## Discussion

Adapting a proven methodology developed by HERG to create videos for other long-term conditions such as asthma, diabetes and epilepsy, we have produced a series of ‘talking heads’ videos that are hosted on the CFHealthHub digital platform to support habit formation with inhaled therapies. These videos extend the multi-component behaviour change toolkit available within CFHealthHub by providing videos of positive role models and allowing CFHealthHub users insight into the experiences of others overcoming the daily challenges of living with CF. In a 607-participant multi-centre RCT, the multi-component CFHealthHub intervention was shown to achieve higher objectively measured adherence to inhaled medications sustained over 12 months, higher body mass index and lower perceived CF treatment burden, without increasing anxiety [7]. The trial was not designed to determine the relative effectiveness of the various intervention components, nonetheless, the ‘talking heads’ videos are an integral component of the CFHealthHub intervention.

There is emerging evidence for the role of peer modelling to increase self-efficacy and promote behaviour change in other long-term conditions. An RCT evaluating videos of patient narratives about their individual struggles and successful hypertension management showed improved blood pressure among people with uncontrolled hypertension [13]. It is thought that the use of patient narratives resonates better among patients and decreases cognitive resistance to behaviour change messages [11]. People with long-term conditions may better identify with fellow patients who are close to themselves and share similar characteristics compared to actors or health professionals conveying factual information. Messages from other ‘people like me’ can help to promote confidence and reduce self-doubt.

‘People like me’ who have successfully managed behaviour change and achieved good health outcomes can also be thought of as ‘positive deviance’. Positive deviance refers to at-risk individuals who have figured things out for themselves and are able to follow beneficial practices that are uncommon within the community, thus achieving better outcomes in a population that shares similar risks [14]. The positive deviance approach has been shown to be successful in inspiring enthusiasm for improvement in other long-term conditions [14].

We found that a key motivation for people with CF who participated in interviews to develop these videos was a desire to help others who may be struggling with similar problems by sharing their experiences. Talking openly about their previous barriers, as well as how they had overcome them, was something they felt might benefit others. In addition to the ‘talking heads’ videos, the content of the interview transcripts also generated valuable insights into difficulties that people with CF often face in motivating themselves and overcoming barriers to adhere to nebulised treatment. Useful strategies were shared such as setting achievable goals coupled with rewards for achievement also the importance of finding a treatment routine that fits around the life of the person indicating the necessity for individually tailored approaches to adherence support.

In our study, there were interesting differences in emphasis when transcripts alone were compared with transcripts viewed alongside video recordings. This discrepancy highlights the importance of developers watching the video in full to ensure all relevant clips are utilised. It may be beneficial for others carrying out similar work to watch the video in full to ensure all relevant clips are utilised. The sample size of this study was limited by budget and time constraints, so we may not have reached saturation in barriers and solutions sampled. While participants using CFHealthHub generally welcomed the video library, some preferred not to view videos to avoid comparison with people like them who have more severe CF or people like them who seemed healthier [15]. Although all participants in the RCT intervention arm had access to the video library, we emphasised that video viewing was optional in recognition that some people with CF prefer to avoid social comparison [16].

## Conclusions

We have developed ‘talking heads’ videos to support behaviour change as part of a complex adherence intervention. The ‘talking heads’ videos aim to increase vicarious self-efficacy with using daily inhaled treatments by showing a range of people with CF managing or increasing their adherence. These videos broaden the behaviour change intervention of CFHealthHub by providing positive role models and allowing CFHealthHub users insight into the experiences of others with CF. Some but not all those who have used the CFHealthHub digital platform have found the videos to be helpful. It is important to sensitively support choice when providing resources aiming to increase vicarious self-efficacy. Our experience may guide the development of similar videos for people with CF and other long-term conditions.

## Appendices

Appendix A: A topic guide for the one-to-one, face-to-face, semi-structured interviews

**Table 5:** A topic guide for the one-to-one, face-to-face, semi-structured interviews.

Topic guide
We are particularly interested to hear about your experiences with nebuliser treatment and to find out more about how you have improved your use of a nebuliser
Part 1: Tell me what it was like when your nebuliser adherence was poor • why was it poor? • did you understand the importance of good nebuliser adherence? • what stopped you from adhering? • what was your health like then? • how did you feel when you weren't adhering?
Part 2: Can you tell me about how you improved your use of the nebuliser? • what prompted you to change? • what strategies did you use to change? • what did you do when things were difficult?
Part 3: I’d like to find out more about how things are for you now. • how are you managing your adherence now? • how is your health? • how do you feel? • what has your improved adherence enabled you to do that you couldn't before?
Part 4: Thinking about your own experience, what advice would you give to other similar people or your younger self with CF? • about how to start to make changes? • what to do when things go wrong/are difficult? • how to keep your motivation going?

Appendix B: Further description of the ‘talking heads’ videos

**Table 6:** Further description of the ‘talking heads’ videos.

Categories	Examples of video descriptions created by the PPI group	Quotes
Advice to younger self	XXX talks openly about the risks of underestimating his condition, missing treatment and how you are the person who can make the biggest positive change	‘If I was talking to my younger self, personally I would say, don’t underestimate your illness and don’t take for granted that you’re ok, because I was ok, but the reason, probably the reason why I was ok is because I was doing plenty of treatment, plenty of exercise, uh, and then as soon as I had to manage that myself, it just kind of dropped off and so you can say that over time you, you’re likely to deteriorate a bit but it does, it has, come hand in hand with not looking after myself as well as I was being looked after when I was a child growing up’
Coping with feeling low	There will always be peaks and troughs with CF, it’s important to keep motivated though	‘…out of the trough, you will come out on the other side and actually it’s just a re-focus, re-focus yourself’
Juggling treatment and life	XXX talks about how seeing adherence data motivates him and supports what he wants to achieve in life	‘if I go in and see my adherence is only 60%, 65% it gives me a bit of a kick thinking, I’m not doing it as well as I should, as well as I think I am, I need to really up it and make sure I am doing things’
Going to University	XXX talks about what motivated him to do his treatment and not rely on IVs	‘When I was a teenager I thought I had a grasp on it, but that’s only because my mum had a grasp on it, and then I quickly realized and kind of leant that I don’t, at all, have a grasp of it’
The importance of nebulisers	XXX discusses thinking long-term and doing your treatment to help you live a normal life	‘If you do adhere and you do use your nebulisers, then maybe you won’t have to have IVs as much, which is a million times worse than nebulisers’
Keeping motivation up	Treatments aren’t a chore, you should look at them like drinking water – just something you have to do	‘it not a chore, it’s helping me, saving me really, and to see it as a chore is the first mistake’
Having a routine	For XXX, the key to doing her treatment is organization and accepting that everyone slips once in a while	‘…in that time read the news, or watch the news or don’t, I try to not associate it with I’m doing my neb, its my 10-15 minute sit down, you know it’s my quiet time…there’s something that isn’t ‘it’s neb time’ because that’s when you feel like it’s taking up your time’
Finding support	XXX shares her views on why it’s important to talk to others with CF	I don’t think anyone will ever understand as much as someone who’s going through the same thing you’re going through …definitely talk to other people who are going through the same thing’
Nebuliser tips	Not cleaning your nebuliser will increase your treatment times	‘…but if you don’t keep it clean and sterile as much as you should, umm, it takes longer because the filters get blocked and then the longer it takes the more, the less likely I am to do, um, to do my nebuliser really, so you’re your own worst enemy in that sense’
Having a normal life	Being a mum with CF, XXX talks about what routine works for her	‘my night time one’s I know will always get done because I’ll do them in bed, kind of enjoy that little extra half an hour in bed as well…’
Being normal	For XXX doing her nebulisers allows her to be well enough to do the fun things in life, rather than just seeing them as a burden	‘…you want to go and do these things or you want to go and enjoy yourself but actually, if you don’t do the neb, you won’t be able to do them anyway …’
Advice to others	XXX talks about how to find a way to make nebs fit into your life	‘use dead time to take your nebuliser, that’s what I would say, and I use that laid in bed in the morning, I love that 5 minutes ‘cause to me that’s an extra 5 minutes in bed when I’m taking my nebuliser’
Late diagnosis	After discovering he had CF later in life (aged 43), the nebulised medicines he started to take completely changed his life for the better. The stark improvement in health after beginning his treatment is all the motivation he needs to take them	‘.…I got the drugs and they started working and it totally changed my life’
Growing up with CF	XXX talks about the positive relationship with his CF team. Even though he was critical of them when he was younger, he reflects that they were only trying to help him	‘If I’d have had access, I think, when I was younger to other people with cystic fibrosis going through what I’ve been through, I think I would have seen straight away, well, what you’re doings’ wrong. You need to be doing your medication, you know it’s easy to be told by everyone or anybody, that you need to be doing something, I think it helps being told by someone whose been through something, you know I’ve had the bad time in my life, I’ve had the decline in my health, um, and if someone had been through that and spoke to me I would’ve really thought about what I was doing’
General tips	XXX provides a tip to help when coughing in a public place	‘I carry a Lucozade bottle around with me, um, and if I cough something up I can make it look like I’m taking a drink from the bottle, but in reality I’m actually spitting out what I’ve just coughed up off my lungs into the bottle …’
Talking to others	XXX talks about the benefit of talking to other people with CF, in being able to offer your experience. Hearing stories from those who are much older with CF has also made him feel much more positive about life expectancy	‘…as I’ve got into my late 20s, speaking to more people it’s just made me a lot more positive on my outlook on life, and it’s given me something to strive for. If I look after myself now, I’ll live longer’
